# “Financial Stability”: A Nightmare for Retired Healthcare Professionals in Southwestern Nigeria: An Interpretative Phenomenology

**DOI:** 10.1177/23779608251350235

**Published:** 2025-06-17

**Authors:** Aderonke Julienne Adetunji, Emelda Zandile Gumede

**Affiliations:** School of Nursing and Public Health, 56394 University of KwaZulu-Natal, Howard College, Durban, South Africa

**Keywords:** financial stability, pension fund, retirement, health workers

## Abstract

**Introduction:**

The transition from work to retirement has been an issue of lively scientific discussion for many and is driven by the constant fear of what is going to happen to their financial stability because of a history of delayed pension payments. Budgetary constraints by the Federal Government led to delays in paying pension benefits. Pension Commission (PenCom) works to ensure adequate budgetary provisions and timely release of funds to reduce the backlog in payments. Many retirees lack proper retirement and financial planning while PenCom continues to emphasize the importance of planning for retirement to ensure financial security.

**Objective:**

This study was conducted to explore the financial stability of healthcare professionals postretirement, in Nigeria.

**Methods:**

An Interpretative Phenomenological Analysis (IPA) design was utilized. The study population consisted of retirees who were 2 years old or older from three tertiary institutions in purposively selected states in Southwestern Nigeria. After obtaining consent, an in-depth interview and focused group discussion (FGD) were used to collect data. Both interviews and FGD were audio recorded, transcribed verbatim, and analysis followed, IPA steps by Smith et al. This was there after being imported into NVivo version 12.

**Results:**

Respondents thought they were financially prepared for retirement, however, the delay of 6 to 15 months before the pension payout was unforeseen. Retirees opted for alternative means of income, like petty trading, Tuck shops, molding blocks, livestock-rearing goats, and working in private institutions. An emerging theme of “Financial instability” and three subthemes: alternate income; flexi-work and exploring hidden talents. Financial security is the key to a peaceful retirement.

**Conclusion:**

Employees need to be prepared for retirement by having multiple sources of funding to enjoy a peaceful retirement

## Introduction

The transition from work to retirement has been an issue of lively scientific discussion for many and is driven by the constant fear of what is going to happen to their financial stability because of a history of delayed pension payments. Issues of budgetary constraints by the Federal Government and Pension Commission (PenCom) straining to ensure the timely release of funds to reduce the backlog ultimately led to delays in the long-awaited benefits. This study aims to provide a rich, in-depth exploration of the lived experiences of retired healthcare professionals in Southwestern Nigeria, focusing on the multifaceted challenges they face in achieving financial stability postretirement.

Retirement marks the withdrawal from active working life. It is a significant transition that impacts individuals both financially and emotionally, and for retired healthcare workers in Nigeria, it represents a critical juncture. Retirement, a phase that promises rest and reflection, often becomes a nightmare for retired healthcare professionals in Southwestern Nigeria. These dedicated individuals, who have spent their lives caring for others, now grapple with the complexities of financial stability during their twilight years.

The mandatory retirement age for public workers in Nigeria is typically 60 years. Retirement is associated with dwindling resources and decreased life satisfaction which may eventually affect the quality of life of the individual (Bonsang et al., 2023). Ugwu and Idemudia (2023) opined that one of the greatest fears expressed by many healthcare workers preretirement is being rendered financially handicapped due to unreliable pension schemes. This situation makes it imperative to extend the work life of the few health workers beyond the age of 60 (Hewko, 2021).

Professionals, transition from their careers, and call for compulsory individual financial planning provided by the employer to ensure economic stability for the well-deserved employees in their retirement years. Among healthcare professionals, retirement is an issue of serious concern, particularly in a post-COVID-19 pandemic experience that left many healthcare facilities with significant shortfalls of health workers (Cutler, 2022). In Nigeria, a significant percentage of nurses were clear about retirement especially those with critical skills that they were not going to exit at 60 and leave their citizens in desperation. This situation called for the government to make amends to employ them as flexi staff.

Retirement financial planning is a critical aspect of ensuring a comfortable and secure life after decades of work. In Nigeria where family structures, social networks, cultural norms, and economic realities intersect retirees face unique challenges and opportunities related to financial planning during their postwork years.

This study utilized an interpretative phenomenological approach to unravel the lived experiences of retired healthcare professionals, to explore financial stability postretirement; through in-depth interviews. This assisted in uncovering underlying meanings and emotions related to financial stability.

## Review of Literature

The retirement process brings physical, mental, financial, anatomical, and functional alterations, which affect the quality of life of older adults (Grassi et al., 2020). To make the adjustment process postretirement more palatable, a retiree receives certain benefits in the form of a gratuity and pension (Soepding et al., 2021).

Pension is an old-age security payment system that provides retirement benefits to pensioners. In Nigeria, broadly speaking, the pension scheme can be categorized into the Defined Benefit Scheme and the Contributory Pension Scheme. The pension system aims to ensure that employees can maintain a basic quality of life upon retirement, with payments managed through government or employer-based contributions. The pension also reflects a commitment from both the public and private sectors to safeguard employees’ welfare, reducing the economic strain associated with old age and unemployment after retirement (Yusuf et al., 2023). The Pension scheme has undergone several reforms to reposition it for better efficiency and effectiveness and to better cater to the welfare of the elderly. From the Pension Ordinance of 1951 to the 2014 Pension Reform Act, several modifications have been suggested and implemented, including the Pay As You Go (PAYG) scheme and the contributory pension scheme. One of the pension reforms that has been initiated in Nigeria is the contributory pension scheme. The scheme serves as a long-term savings plan to ensure that retirees have financial security after leaving the workforce (Faronbi et al., 2021). However, the implementation of this pension scheme leaves very little to be desired as many retirees as possible, including those in the Nigerian pension system, while evolving, still grapple with inefficiencies, delays, and bureaucratic hurdles. Retirees often encounter challenges accessing their pension funds in time (Faronbi et al., 2021).

Nigeria has long experienced poor pension administration which has resulted in incessant complaints among retirees about nonpayment of their pension. This situation justifies their dissatisfaction despite the various reforms and new laws targeted at improving the pension administration (Ukwu et al., 2023).

The setbacks of the Contributory Pension Scheme include the lack of prompt remittance of the contributions made by both employers and employees; lack of easy access by pensioners to their benefits as at when due, and lack of prompt verification process as well as corrupt practices which culminate to embezzlements (Ukwu et al., 2023). The Nigerian pension system, while evolving, still grapples with inefficiencies, delays, and bureaucratic hurdles. Retirees often encounter challenges accessing their pension funds in time (Faronbi et al., 2021).

Many Nigerians lack adequate financial literacy to develop a retirement savings plan (Nam & Loibl, 2021). A significant contributing factor is a lack of trust, stemming from a history of systemic failures of retirement plans by the various Nigerian governments. Consequently, retirees have lost interest in the government-initiated pension scheme due to delays and refusals of pension entitlements, which adversely affect their quality of life (Adewumi, 2024).

Financial literacy retirement planning encompasses the knowledge and skills needed to make informed financial decisions (Kapoor, 2024). It plays a pivotal role in retirement planning. Weakness in financial literacy often leads to inadequate retirement funds, leaving retirees struggling to meet their financial needs (Nagaprakrash et al., 2023). Despite government initiatives to promote financial savings for retirement, gaps in financial literacy persist within the retired healthcare workforce (Chen & Chen, 2023). Financial illiteracy is responsible for poor decision making by retirees, posing a wide variety of challenges in their retirement life, thus leading to anxiety, depression, and compromised wellbeing. The retirement scheme adversely impacts other areas of their existence, including their health and mental wellbeing and quality of life postretirement (Kettlewell & Lam, 2022). In Nigeria, the health sector laments the poor remuneration and reward system in the country (Aliu et al., 2023). Healthcare workers are not immune to these realities, as evidenced by the mass exodus of healthcare workers in search of better economic rewards for their skills and expertise (Kettlewell & Lam, 2022; Handley et al., 2021).

Over the years, the financial stability of healthcare professionals in Nigeria has encountered significant hurdles leading to low job satisfaction and demotivation. In recent times, the challenges persist, with many healthcare workers still contending with payment upgrading that is commensurate to their work demands and output, which results in occasional industrial action while making their demands known to the Federal government. Despite ongoing efforts to enhance the healthcare system, the financial burden on healthcare professionals due to delayed payment remains a major concern. However, it is only those who have prior investments that are self-sufficient and can live better as they desire (Chen & Chen, 2023; Kapoor, 2024); whereas those who do not have investments with no good savings live poorly with a lower quality of life. Lightman and Akbary (2023) revealed that in Canada, compared to other immigrant women, care worker women had lower expected total income and faced downward mobility after retiring; this is because they were less likely to contribute to private pensions before retirement and more likely to rely on public pensions afterward.

The global nursing shortage has contributed to the retention of older nurses in the workforce Sudharsanan and Bloom (2018). In a study conducted in China, Liu et al. (2018) found that while many older nurses experience delayed retirement, few have concrete retirement plans—an outcome influenced by factors such as work environment and job satisfaction. Nonetheless, effective nurse–patient communication and opportunities for personal and professional development were identified as facilitators of retirement planning. Studies suggest that delayed retirement may have positive health implications for older adults, especially in settings with underdeveloped health insurance and pension systems. For instance, in countries like China, where many older individuals lack employment-based health coverage or continue contributing to pension schemes beyond the statutory retirement age, late retirement can serve as a vital substitute for formal social security (Li et al., 2024).

According to Zhan et al. (2022), an important obstacle facing many older workers is ineffective transitioning to retirement. Utilizing the resource-based dynamic model of retirement adjustment, the study revealed that preretirement resources such as physical health, mental health, financial wellbeing, family support, etc, affect the retirement process. Yuan et al. (2022) opined that working later in life can help reduce depression, especially for those who don't have employment-based health insurance or are still paying into a pension. Markowski et al. (2020) emphasize the importance of fostering a supportive organizational environment that not only respects retired nurses who wish to remain professionally active but also attends to their individual and professional needs.

In another study on the predictors of retirement anxiety, Ogunsemi et al. (2023) observed that 70% to 85% of Nigerians suffer from retirement fear. Most Nigerian workers dread life after retirement (pensioners) because the future appears bleak, and a lot of challenges are encountered, which may be physiological, psychological, and economic (Gureje et al., 2008). There is a need to provide comprehensive guidance information relating to emotional, financial and other aspects of the retirement processes in collaboration with employers. This support through training and conferences will provide therapy for elderly workers (Omojugba, 2024).

There is limited research on the financial stability among retired healthcare professionals in Southwestern Nigeria. One of the greatest fears expressed by many healthcare workers in connection with their retirement is being rendered financially handicapped due to unreliable pension schemes for retirees (Ugwu & Idemudia, 2023).

The researcher embarked on the interpretative journey and recognized that financial stability is not merely about numbers, but is intertwined with dignity, self-worth, and the ability to lead a fulfilling postretirement life. Delving into their narratives, it is hoped to inform policy recommendations and improve pension systems, financial literacy programs, and social support initiatives. Amplifying the voices of retired healthcare professionals, the researcher hopes to contribute to a more holistic understanding of retirement planning and enhance the wellbeing of future retirees.

### Theoretical Framework Underpinning the Study

This study employs Robert Atchley's Continuity Theory of Ageing (1970s) as its theoretical framework, which posits that individuals experience optimal aging by maintaining continuity in activities, relationships, and personal identity. The theory emphasizes the importance of both internal and external continuity—retaining self-concept and social roles rooted in past experiences—to promote stability amid significant life transitions such as retirement, health challenges, and evolving family dynamics (Atchley, 1989).

By applying this framework, the study examines the retirement experiences of healthcare workers, offering a nuanced analysis of how personal histories and social contexts shape adaptation strategies (Henning et al., 2016). The Continuity Theory provides a structured lens through which the multifaceted nature of retirement can be explored, including psychological and emotional adjustments, changes in social relationships, and shifts in familial and communal roles. This approach facilitates a deeper understanding of the meanings healthcare professionals assign to retirement and the extent of their preparedness for this major life transition, addressing gaps in the existing literature and contributing to a more holistic comprehension of the ageing process.

## Methods

### Research Design

This study adopted the Interpretative Phenomenological Analysis (IPA) to explore the retirement experience of healthcare professionals as meant to them in their world postretirement (Smith et al., 2009; Creswell & Poth, 2023). This mode of inquiry allowed the retired healthcare professionals to provide meaning to their world of retirement, while the researcher ascribed more understanding and interpreting the participants’ journey (Smith et al., 2009). With the complexity of the retirement experience and the significance that retirees attach to it, the IPA technique is best suited for this study. The human experience can also be examined by the IPA from both its perspective and its mode of occurrence. Humans are “sense-making creatures,” according to IPA researchers, a participant's explanation of an event will likely reflect their attempt to make sense of what they have experienced. Additionally, IPA is idiographic, meaning that it is dedicated to thoroughly examining every case or event, to fully understand everyone's unique experience and the meaning they are constructing of it (Smith et al., 2009).

### Research Questions

What are the factors responsible for financial instability and the nightmare of retired healthcare professionals?

### Study Population and Sample

The study population consists of retirees who are 2 years and above postretirement from three selected Federal teaching hospitals in South-Western Nigeria. A purposive, nonprobability sampling technique was utilized to recruit participants based on their understanding of the research problem and willingness to participate. A total sample of 21 respondents was recruited for the in-depth interview (IDI) and 22 respondents were recruited for the focused group discussion (FGD). The sample comprised both males and females from the selected Federal teaching hospitals from the three states under the study. All healthcare professionals and allied workers within a health fraternity were part of and respondents were recruited based on their availability and willingness to participate in the study. A total of 21 in-depth interviews (10 from Lagos state, 5 from Oyo state, and 6 from Ogun state) were conducted until data saturation was reached. The FGD of three groups was also conducted in each state to enrich the data namely, Lagos 8, Oyo 7, and Ogun 7.

### Inclusion/Exclusion Criteria

The inclusion criteria were Federal health institution retirees who were 2 years and above and were willing to share their experiences. The exclusion criteria were retirees below 2 years of retirement; and with a history of chronic depression or mental disorder. This is to ensure the reliability and correctness of information.

### Data Collection Process

#### Phase 1: In-Depth Interviews

Permission to conduct the study was obtained from BREC at the University of KwaZulu-Natal (UKZN). Access to each facility was facilitated through gatekeepers, who are the executive members of the National Association of Federal Health Pensioners of Nigeria. An official letter from the National Headquarters of the Association introduced the researcher to potential participants during their meetings. Subsequently, interested individuals voluntarily indicated their willingness to participate in the study.

Arrangements for data collection were finalized with the participants at a time and venue convenient to them. The researcher was assisted by two research assistants throughout the data collection process. All interviews were conducted in English. Probing was done until data saturation. All interviews were recorded with the permission of the participants, and the data collection process among the three states of Nigeria took a period of 7 months (January 2022 to July 2022). A total of 21 participants formed part of the in-depth interviews. To ensure credibility, prolonged engagement during interviews was ensured, each interview lasted between 40 and 60 min. In qualitative study, data collection and analysis go hand in hand. The researcher transcribed the data verbatim and then analyzed using IPA by Smith et al. (2009). The themes that emerged were grouped according to the retiree's financial instability and lived experiences (Table 1).

**Table 1.** Biographic Details of the Participants for Phase 1 of the Study.

Participants’ gender and age in years	Number of participants per phase of the study	Specializations of the participants	Years of experience postretirement	Country Nigeria (state-specific)
F (61 years)	21	Nurse	3	Lagos
F (62 years)	21	Nurse	6	Lagos
M (63 years)	21	Medical Laboratory Scientist	3	Lagos
F (61 years)	21	Nurse	3	Lagos
F (63 years)	21	Community Health Consultant.	5	Lagos
M (63 years)	21	Electrical Engineer	3	Lagos
M (63 years)	21	Administrator/ Auditor	4	Lagos
M (63 years)	21	Confidential secretary	3	Lagos
M (63years)	21	Nurse	3	Lagos
M (55 years)	21	Health Information Officer	3	Lagos
F (68years)	21	Nurse	8	Oyo
F (68 years)	21	Health Assistant	13	Oyo
F (61years)	21	Nurse	4	Oyo
F (64years)	21	Radiographer	7	Oyo
F (64years)	21	Nurse	4	Oyo
F (66years)	21	Nurse	12	Ogun
F (63years)	21	Nurse	7	Ogun
F (55years)	21	Nurse	6	Ogun
F (64years)	21	Nurse	4	Ogun
F (58years)	21	Nurse	7	Ogun
M (68years)	21	Anaesthetic technician	8	Ogun

Matrix table for biographic details of the IDI participants.

#### Phase 2: Focus Group Discussion

Qualitative research used many methods for data collection, the researcher also embarked on focus group discussions to complement data collected through in-depth interviews. Participants with almost similar experiences and who met the eligibility criteria were purposefully asked to partake in the second phase of the data collection process. Some of the participants were new to the focus group discussions and were not available for the first phase because of work commitments. An information sheet with the details of the next phase was given and consent was obtained before participants engaged with the study. The researcher was assisted by two research assistants in this phase to collect data in the three states of Nigeria (Lagos, Ogun, and Oyo). Venues and times were chosen to suit the participants’ availability. A total of 22 participants took part in the Focus group discussion, they were given codes to ensure anonymity throughout the study. Focus group discussion was conducted in English.

At this stage, no sensitive issues were discussed, the researcher wanted to engage the retiree on common strategies used whilst waiting for their pension payout. Quite a lot of information was gained in this phase, as they share different coping mechanisms, like having a spaza shop and livestock rearing to gain money, and some resorted to engaging in flexitime work in private institutions to earn a living. Probing survival strategies helps to empower each other with means of getting an extra income, as the pension payout will not meet all their basic needs. Data collection and probing continued until data saturation. Data was transcribed verbatim and then analyzed using IPA by Smith et al. (2009) (Table 2).

**Table 2.** Biographic Details of the Participants for Phase 2 of the Study.

Participants’ gender and age in years	Number of participants per phase of study	Specializations of the participants	Years of experience postretirement	Country Nigeria (state-specific)
F (66 years)	22	Secretary	7	Lagos
F (62 years)	22	Nurse	2	Lagos
M (67 years)	22	Nurse Educator	10	Lagos
F (63 years)	22	Nurse Educator	3	Lagos
F (63 years)	22	Community Health Consultant.	3	Lagos
M (72 years)	22	Engineering Technician	13	Lagos
M (63 years)	22	Nurse	3	Lagos
M (63 years)	22	Confidential Secretary	3	Lagos
F (72 years)	22	Nurse Lecturer	13	Oyo
F (69 years)	22	Nurse Lecturer	11	Oyo
F (62 years)	22	Nurse	3	Oyo
F (67 years)	22	Nurse	8	Oyo
F (68 years)	22	Nurse	10	Oyo
M (68 years)	22	Engineering Technician	15	Oyo
M (68 years)	22	Engineering Technician	15	Oyo
F (66years)	22	Nurse	12	Ogun
F (63years)	22	Nurse	7	Ogun
F (55years)	22	Nurse	6	Ogun
F (64years)	22	Nurse	4	Ogun
F (58years)	22	Nurse	7	Ogun
M (68years)	22	Anaesthetic Technician	8	Ogun
M (64 years)	22	Health Assistant	6	Ogun

Matrix table for biographic details of the participants.

### Data Analysis

Data from the study were analyzed using IPA following steps as indicated by Smith et al., (2009) (Table 3). All interviews were transcribed verbatim. Data were analyzed using IPA following the six recommended steps and thereafter imported into NVivo version 12 to manage and organize the data. Anonymity was ensured by giving participants codes and no personal details were captured during the interview. Findings were reported along the themes and subthemes identified (Table 4).

**Table 3.** The Six Steps of Data Analysis by Smith et al. (2009).

Phases	Procedure
Phase 1	The researcher immersed in the data through reading and re-reading transcripts to become familiar with the participant's account
Phase 2	Repeated reading allowed an understanding of how certain sections of the interview fit together with initial notetaking
Phase 3.	Data was imported into NVivo version 12 to manage and organize the data. Developing emergent themes
Phase 4	Searching for connections across emergent themes; clustering the themes more analytically to produce a superordinate theme. The process involved an iteration that utilized a deductive reasoning approach Tables were generated of themes using identify verbatim (text) to indicate each theme
Phase 5	Analysis of other cases (within-case analysis)
Phase 6	Looking for patterns across cases and generating a coding frame

**Table 4.** Theme and Subthemes of Their Experiences of Financial Stability.

Themes	Subthemes	Meanings
**Financial instability**	1. Alternate income	Activities that can increase income such as setting up a small business, and petty trading, etc
	2. Flexi-work	Ad hoc engagements that resulted in monetary gain at the convenience of the retiree
	3. Exploring hidden talents	All discoveries postretirement that strengthened financial stability and brought about satisfaction

The six steps identified by Smith et al. (2009) were followed as stated in Table 3.

## Results: Emerging Themes

This study explored the factors that led to financial instability and the source of nightmares of retired healthcare professionals. Based on the respondents, an emerging theme “Financial instability” was noted and three subthemes were: alternate income, flexi-work, and exploring hidden talents.

### Theme: Financial Instability

A prevalent factor for individuals not making adequate retirement plans is a lack of financial knowledge, which could lead to financial instability. Financial knowledge is one of the components of financial literacy, which is understanding financial ideas to enable individuals to make well-informed decisions. This includes having ideas on the time value of money, debt management, savings, and investments. Even though individuals may not know how much to save to enjoy a comfortable retirement, but knowing how best to invest money can be a good source of financial stability (Antoni, Saayman & Vosloo, 2020). This study revealed that all the respondents had prepared their minds to embrace retirement, they were aware of the decrease in the monthly payout, but the delay just spoiled it all. Some of the respondents knew that retirement comes with a dwindling economy and would have loved to better prepare before it arrived, since the immediate stoppage of a regular income postretirement plunges the retired healthcare professional into the reality of an economic crisis.

As such this theme outlines how the retirees try to manage the situation pending the unstipulated time that the pension payment is made as stated by the respondents:

*I know I am going to retire, I know so I was working towards it, but there is no money, don’t let me tell you lies, there was no money, I was the only one carrying the financial burden… my children went to private school, you know what it means, so all the money were going towards that side, the project we were doing, up till now we are not able to complete it, that is why we are still here. So, no money to do anything … I have the idea of getting a shop, for petty trading …. a small chemist, but there is no money for doing anything for now o. (F, IBA 02)*

*“It took a whole year. I left service in June 2019. The payment came in June 2020. So, after 2020 we started receiving that small token that they started giving us as pension money.” (F, LAG 02)*

*“Unfortunately, when we reti retired, we were the first set to experience this untimely paid gratuity, it was another case, but it was God's grace that kept me going till date because we were unable to be paid until we carried placards.” (F, ABK 02)*

### Subtheme 1: Alternate Income

Retirees who participate in a variety of postretirement activities are better able to adapt to changes in their surroundings and social life (Adetunde et al., 2016). These part-time jobs and businesses could be micro, small, and medium enterprises (Lloyd & Robbins, 2014). Some of the retirees initially were confident that they may not have issues waiting for the payment of their pension. They probably thought that their savings might be adequate before their pension payment materialized. Thus, some found not to be so as expressed below, and with support from their spouses, had to commence on an alternate business. This study also revealed that some of the respondents were offered postretirement jobs which they embraced, even though the respondents had made financial plans before retirement.

*Also, before the lump sum was paid, somebody offered me a job in a hospital to just put things together for them and to put some nurses through, based on my experience in the profession, which I accepted… now coming back home late with the situation in Lagos, most especially the traffic jam, so I had to quit the job after a while, so I just faced my shop business. (F, LAG 02)*

*… about 6–7 months when the real issue was now done on me. So, what I just did in my case was to go back to the water business, and to the glory of God, the water business is doing well with the help of my wife, we are doing well with that. But I must confess, retirement life is not an easy thing, you know you have been earning salaries before, and suddenly you are no longer earning, and the… (M, LAG 09)*

*But ever since I retired to the glory of God, I can go up and down and have a small business of my own that I manage. (F, ABK 02)*

*I am into so many other businesses that can give me money, like cryptocurrency and whatever is legitimate that can give me money. (Laughs) (M, LAG 03)*

### Subtheme 2: Flexi-Work

Seasoned professionals after years of experience working in traditional settings may find the allure of a flexible work schedule particularly alluring. For retirees in particular, flexible employment may be extremely beneficial financially and provide freedom of choice to those who choose to utilize it (Holborn, 2022).

Being retired can become a bit boring, especially if the retired person stays at home all day without anything to break the monotony. A nonfinancial advantage of flexi-retirement is having a flexible position that the retired persons enjoy working at, creating their schedule, and working on projects that could be exciting, enjoyable, and improve their general happiness and well and wellbeing.

In this study, a few of the respondents had the opportunity of being invited to participate in flexible work. The work still allowed them to fix their schedule to take part in the work for specific days of the week and still do other engagements. Flexible working is an arrangement that allows employees to set their working hours and patterns. Working from home, part-time, or compressed hours are some of the options available. This included but was not limited to private consulting services in other facilities, which could still give them a sense of fulfillment after retirement, or taking part in medical research that is for a specific period, as stated:

“*Presently I am just consulting. (Laughs) I told my colleagues that I am available for consultation, and based on my professional experience, I offer professional advice, yes, that is my vocation for now” (M, LAG 03)*

*After, I returned from my daughter's place, one of the Doctors approached me if I would like to participate in a research project, considering my years of experience in the oncology unit, and I accepted. (F, LAG 01)*

*“As young as I am, am not finding it too easy to cope with altered income, I have been earning close to 500,000 naira (R 7,858) before, and, this time you are now asked to be earning 100,000 naira (R 1,571) … because I still love the job that I am doing” (F, ABK 03)*

### Subtheme 3: Exploring Hidden Talents

As people leave longer-term traditional professions, many are attempting to determine whether to retire, reboot, or re-wire themselves. Increasingly, people must work longer hours or work part-time during their retirement due to longer life expectancies and more years in retirement (Rappaport et al., 2020).

In their spare time, senior citizens can engage in volunteer work, passion projects, and hobbies that allow them to utilize untapped talents. A lot of people take classes to pick up new abilities. In this study, many of the respondents were energetic and full of vigor to take up new challenges, instead of waiting for their pension payout. Some are already employers themselves, hiring people to work in their small to middle-scale businesses. This is expressed as follows:

*I am a block molder. I mean concrete block, So, I must work almost 24 hours, to make sure that nothing crumbles my business. I must be on the neck of other workers that I have employed to mold blocks for sale as a source of income to supplement the salary, you do this, you have not done that, where is the driver, why customers are complaining, non-delivery made today? Have they gone to buy fuel? So, you know, it has been but then the joy of this is, this is your own, having your own business. (M, ABK 06)*

*I am into livestock, I am farming, so that is what I am doing presently…* *farming was my father's job and is one thing I love doing. We were all brought up to cherish farming. It is a good way of supporting one's family. It just occurred to me one day that I could venture into the business, I then approached the manager of the factory of my intention, and I enrolled for an apprenticeship, and since then, I have continued. (M, LAG 08)*

*Immediately after I retired, I saw the need to go back to my father's homestead, where he owns huge acres of unused land, I started the farming business, and with a little bit of this and that, it ended up being popular for livestock like cows, sheep, goats, and chicken. The money that I make per day, is not even close to what I was earning whilst fully employed, one man's meat is indeed another man's poison. I am considering grooming my son, so that he becomes an entrepreneur at an early age, unlike me. (M, LAG 03)*

The researcher has presented the theme and subthemes that emerged in this discussion, supported by a few of the relevant incepts from the respondents. It is pertinent that retirees should stay active doing what they enjoy doing even at retirement, especially activities that would bring about financial stability which will ultimately reduce stress and enhance wellbeing.

## Discussion

Drawing on the IPA approach, this study highlighted the financial instability of all healthcare professionals postretirement in Nigeria. Numerous global studies underscore the significance of financial literacy in the current period, as it has been identified as a critical ability that fosters sound financial attitudes in recent times (Achari et al., 2020).

Antoni et al. (2020) cited Willows (2019) study, which revealed that the majority of South Africans will reach retirement age with insufficient retirement income, stating that one possible explanation for this could be that customer financial illiteracy and poor insight into the importance of retirement savings plan per proportionate age to enjoy a comfortable retirement. This is further concurred by Dhlembeu et al. (2022), who stated that the shift in pension and retirement planning has a dire effect on individual employees.

Contrary to the above Stattin and Bengs (2022), declare that some employees are still fit, for purpose and, thus should not be forced to exit because of age, because this economic crisis, they are facing, could be prevented by having fewer employees exiting the system, hence needing huge sums of money to pay in each financial year.

When it comes to financial security, wealth plays a bigger role as people age. The wealth that people and households accumulate over the course of their lives—both financial and material assets, such as homes and land areas to fund consumption and protect them against shocks when they retire (United Nations, Department of Economic and Social Affairs, Population Division, 2020).

The United Nations reports that, in high-income nations, adults save over 46% of their income for retirement, compared to only 16% in middle and 38% low-income nations (World Social Report, 2023). These disparities reflect people's varying capacities to accrue wealth based on their incomes.

Differences in financial stability exist between healthcare professionals who have been retired for more than 10 years and those who have been retired for less than 10 years. This disparity arises from salary reviews and the lack of proper follow-up, resulting in pensions based on outdated salary structures. Those who retired from Federal government institutions generally see their pensions adjusted with salary reviews. In contrast, retirees from state institutions, especially under noncontributory pension schemes, often face embezzlement issues without proper oversight, leading to a significant decline in their quality of life. This issue is particularly pronounced for retirees without grown children who can provide financial support. However, retirees with grown, employed children tend to receive better support, improving their financial stability.

A lack of financial literacy limited digital capabilities, illiteracy in general (especially in developing countries), discrimination, and the absence of financial services, products, and digital infrastructure affect some groups of people more than others.

### Alternate Income

The respondents expressed dissatisfaction and anxiety when their emoluments and pensions were delayed, payment period was between 6 and 18 months postretirement. This supports the findings of Ugwu and Idemudia (2023) which revealed that a long delay in payment of retirement benefits could heighten the feeling of anxiety among retirees. Söderbacka et al. (2020) also opined that owning other business investments was significant in reducing the shock and anxiety associated with the retirement of healthcare workers. Despite the initial preparations of personal savings, many of the respondents resulted in alternate income such as petty trading, and tuck shop ownership, molding bricks for public sales, all putting some food on their tables, while waiting for pension payout. These statements are supported by Faronbi et al. (2021), whose study revealed that over half of the participants (53.7%) were involved in some sort of economic activity, with the majority operating small-scale enterprises (petty commerce with less than $135/ R 2,552).

### Flexi-Work

Flexi-work according to the Cambridge dictionary (2023) “is a situation in which an employer allows people to choose the times that they work so that they can do other things.” Flexible work arrangements play a crucial role in supporting retired healthcare workers as they transition into a new phase of life. These arrangements allow for greater work–life balance, accommodate individual needs, and enhance overall wellbeing. Healthcare workers can negotiate flexible schedules that accommodate their preferences. Adjusting start and end times or working nontraditional hours can enhance work–life balance (Clendon & Walker, 2016).

### Exploring Hidden Talents

Faster Capital (2024) revealed that it's normal to feel empty when a career you've spent decades pursuing comes to an end, but this time also offers a rare opportunity to discover new hobbies and passions that may have been neglected during the working years. The ideal time to pursue long-held goals that were postponed due to employment obligations is during retirement. However, in the case of retirees, it is the financial instability that came with the delay in the pension payout of their salaries (Braturn & Asaba, 2021). One exciting finding from this study is that some of the respondents branched into farming and are now employers of labor, creating jobs for the unemployed. The businesses that started as small have now metamorphosed into small-medium based. They now manage both human and material resources, meaning that the hard times have made them stronger financially and have become entrepreneurs.

## Strengths and Limitations

The researcher gained a comprehensive understanding of the experiences of retired healthcare professionals. Using the IPA to analyze such experiences, gave insight because it has assisted in gaining a deeper understanding of the nightmare the retirees go through financially before their retirement benefits are paid to them; the information was elicited from the primary source. The retires share their coping skills, that is benchmarking skills acquired through the process, and unearthing hidden talents which gave rise to employment opportunities for other community members was a tremendous achievement.

The limitation of the study is that the views of the retirees were only captured after they exited the system. Such information should be shared while fully employed to be well prepared for this dilemma. The views of financial managers, policymakers, and as well as pension scheme management were not sought, to explain the reasons behind this notorious delay in pension payout. It is believed that if they knew the frustration they cause to the senior citizens, who have served the country with passion and dignity, maybe they would change their attitude and work ethics in dealing with processes related to pension payout for the retirees.

## Implications for Practice

The research findings will inform the hospital administrators and the pension administrators about the difficulties experienced by retirees in accessing their pension payout. They need to upgrade their systems to be efficient and meet the best practices for the effective transition of health workers to retirement. Benchmarking with other companies or neighboring countries can be the best source of information and establish a partnership for the best client services. Inclusive planning for the effective, seamless retirement of deserving employees is the key.

## Conclusion

The findings of this study revealed that retirees had to look for alternate sources of income to survive while waiting for their pension payments. Younger healthcare retirees often support older retirees by providing care for those whose children are abroad or distant. These children frequently pay stipends to caregivers who check on their parents’ wellbeing. In communities with elderly residents, younger healthcare workers visit daily or weekly to assist those with health conditions like arthritis or other ailments, receiving stipends for their services.

Other alternate sources of income include: Retirees staying in their own homes can also babysit for young couples who have regular jobs, earning stipends for looking after such babies until the parents return from work. Additionally, retirees might engage in organic farming to grow vegetables, enhancing their quality of life and providing a source of income. Part-time jobs such as coaching, counseling, or mentoring younger individuals also allow retirees to stay active and pursue personal interests while earning extra income.

Many are dissatisfied with the current pension scheme operating in Nigeria. They also expressed views indicating their sadness at being neglected by a government they had served for a considerable part of their active years. The implication of this is that many retirees experience increased anxiety, and panic attacks, because of being not financially, emotionally, psychologically, and socially empowered to deal with life as retirees. We recommend that the present pension scheme should be accompanied by robust retirement plans during the last decade of retirement. The Nigerian government and employers of labor should be proactive in preparing the older members of the workforce for the challenges associated with retirement, one of the most significant being financial insecurity. Retirees are senior citizens, and the government should be concerned with their welfare and wellbeing, to avoid creating a negative impression of Nigerian society.

## Supplemental Material

sj-docx-1-son-10.1177_23779608251350235 - Supplemental material for “Financial Stability”: A Nightmare for Retired Healthcare Professionals in Southwestern Nigeria: An Interpretative Phenomenology Supplemental material, sj-docx-1-son-10.1177_23779608251350235 for “Financial Stability”: A Nightmare for Retired Healthcare Professionals in Southwestern Nigeria: An Interpretative Phenomenology by Aderonke Julienne Adetunji and Emelda Zandile Gumede in SAGE Open Nursing
