# Supplementary material for: “Financial Stability”: A Nightmare for Retired Healthcare Professionals in Southwestern Nigeria: An Interpretative Phenomenology
Source: SAGE Open Nurs. 2025 Jun 17;11:23779608251350235. doi: 10.1177/23779608251350235 (PMC12214323; doi:10.1177/23779608251350235)
Supplement: sj-docx-1-son-10.1177_23779608251350235 - Supplemental material for “Financial Stability”: A Nightmare for Retired Healthcare Professionals in Southwestern Nigeria: An Interpretative Phenomenology [file sj-docx-1-son-10.1177_23779608251350235.docx]

**INTERVIEW GUIDE**

**General Information**

**Section A (**Demographic data)

Site ………………………………Age ………….. Sex……..

The institution retired from -------------------------------------------------------

***Section B Key questions***

1. Please introduce yourself (Demographic profile of participants: probe age, sex, occupation, and how long you have been retired)
2. What does retirement mean to you?
3. How would you describe your experience of retirement *(probe before, & now)*
4. How have you been coping with retirement*? (Probe lifestyle; daily routine; self-care: enjoyment)*
5. How prepared were you before retirement? [*Probe plans related to housing, access to health care services, your finances before gratuity was paid, issues related to the ability to cope generally, was there any benefit to you?]*
6. How would you describe the changes in your life since retirement*? (Probe social, etc)*
7. What are the challenges/problems confronting you since retirement*? Probe common health challenges, social issues, and abuses with you as a retired person.*
8. How have you been coping with retirement*?* how would you describe your feelings

*[probe spending leisure, feeling fulfilled in life, etc)*

1. How would you describe the changes in your life since retirement*? (Probe Psychological, and spiritual aspects, positive and negative effects, etc)*
2. What are the challenges/problems confronting you since retirement*? Probe common health challenges, psychological, and physical, abuses with you as a retired person.*
3. What efforts/strategies have you made to deal with the challenges?

*(probe health care seeking behavior, access to PHC services, private facility, spiritualist hospital admissions experiences, issues relating to hospital bills, sources of medication & their sources*

1. Based on your experience, what tools are required to promote a healthy lifestyle and access to care among pensioners in Nigeria (*probe algorithm of care, health maintenance strategies, health insurance schemes)*
2. How would you like the retirement to be arranged in Nigeria

*Is there any other thing that we have not touched that you would like to expatiate on?*

THANK YOU

**Focus Group Discussion (FGD) guide**

**Section A: General information**

Site -------------------------------------------------------------------------------------------Time --------------- Date ----------------------, Place/ Venue-------------------------

Number of participants……………...

Title of the study: Developing guidelines to enhance the quality of life post retirement: A South Western Nigerian Study.

**Section B:**

**1.** Please discuss the measures that the government should put in place to enable comprehensive access to health care services for retirees.

*This focus group discussion will be facilitated through the interactive communication skills that involve probing, validation, paraphrasing and summarising*

THANK YOU
